# Boarded patients, blurred benchmarks: an opportunity for CMS

**DOI:** 10.1093/haschl/qxag226

**Published:** 2026-08-25

**Authors:** Elyssa F L Grogan, Natalia Sifnugel, Arjun K Venkatesh, Ula Hwang

**Affiliations:** Department of Emergency Medicine, NYU Grossman School of Medicine, New York, NY 10016, United States; Department of Emergency Medicine, NYU Grossman School of Medicine, New York, NY 10016, United States; Department of Emergency Medicine, Yale School of Medicine, New Haven, CT 06519, United States; Department of Emergency Medicine, NYU Grossman School of Medicine, New York, NY 10016, United States; Geriatric Research, Education, and Clinical Center, James J. Peters VA Medical Center, Bronx, NY 10468, United States

**Keywords:** boarding, emergency care, quality, access, safety, age-friendly care

## Abstract

The Centers for Medicare & Medicaid Services recently introduced 2 clinical quality measures that intend, in part, to disincentivize hospital boarding: a patient safety concern that involves holding admitted patients in the emergency department while an inpatient bed is unavailable. The Age-Friendly Hospital Measure (AFHM), a structural measure, asks hospitals to attest to having protocols in place to reduce boarding for older patients. The Emergency Care Access and Timeliness (ECAT) measure, an intermediate outcome measure, will require hospitals to report on the proportion of boarded patients. Each measure was developed under a different parent quality reporting program—1 inpatient and 1 outpatient—which has the unintended consequence of producing meaningful variation between the measures. We describe opportunities to standardize and synergize the AFHM and ECAT to capture the prevalence of boarding uniformly, prevent gamesmanship, and enhance their impact on hospital performance. In particular, we highlight the importance of stratifying ECAT by the older-adult patient population to complement AFHM and since older adults are uniquely vulnerable to boarding and its associated safety risks.

Key takeaways

- Hospital boarding is an endemic quality of care and access issue for patients admitted from the emergency department.
- CMS has sought to incentivize hospitals to prevent excessive boarding by introducing 2 clinical quality measures that address boarding in part: the Age-Friendly Hospital Measure and the Emergency Care Access and Timeliness Measure.
- CMS should consider cross-coordinating these measures by (1) positioning both for inclusion in performance-based payment programs, (2) adopting a uniform definition of a boarded patient and boarding's threshold for harm, and (3) including a stratification of older adults in boarding reporting to highlight this vulnerable population.

## Hospital boarding

Boarding, holding admitted patients in the emergency department (ED) while an inpatient bed is unavailable, is endemic and continues to worsen nationally despite being preventable. It is observed in the rooms and hallways of the ED, but is caused by an acute health care system constrained by operational and capacity limitations both upstream and downstream of the ED. Its impact reaches patients as lower care quality and increased risk of poor outcomes. Older adults bear the greatest burden—they board more than younger adults and are more vulnerable to delirium, functional decline, and other iatrogenic harms of boarding.^1,2^ Ensuring timely and safe care for all patients requires a concerted effort to mitigate boarding.

## Regulating boarding

As the largest payor to US hospitals, the Centers for Medicare & Medicaid Services (CMS) maintains a portfolio of 500+ active quality measures across health care settings tying reporting and performance to reimbursement for health services. Two recently developed quality measures target boarding: (1) the Age-Friendly Hospital Measure (AFHM) and (2) the Emergency Care Access and Timeliness (ECAT) Electronic Clinical Quality Measure (eCQM) (Table 1). As the ED sits at the crossroads of outpatient and inpatient care, these measures incidentally belong to separate programs: the Hospital Inpatient Quality Reporting Program (HIQR) and the Hospital Outpatient Quality Reporting Program (HOQR), respectively. The CMS has multiple strategies seeking to address prior gaps in measurement, including strengthening integration across measures and closely evaluating the impact of measures on institutional goals. These boarding measures pose another opportunity to address these gaps and achieve sustainable quality improvements.

**Table 1.** Comparison of CMS quality measures related to hospital boarding.

	Age-Friendly Hospital Measure (AFHM), domain 3, statement 4	Emergency Care Access and Timeliness (ECAT) electronic clinical quality measure, component 4
CMS parent program	Hospital Inpatient Quality Reporting (HIQR)	Hospital Outpatient Quality Reporting (HOQR)
Consensus-based entity endorsement	Not endorsed	Endorsed
Type	Structural	Intermediate outcome
Reporting requirements	Attestation of “yes” or no” to having protocols in place to reduce either ED length of stay to <8 h or boarding to <3 h in a targeted percentage of older adults	Proportion of all ED visits resulting in admission where the patient boarded for >4 h
Threshold of harm	3 h	4 h
Definition of boarding time	Time from Decision to Admit order to ED departure for admitted patients; no specific guidance on how to define decision to admit	Time from Decision to Admit order to ED departure for admitted patients Decision to admit defined as earliest of 4 time points: An evaluation that results in a decision to admit An order to admit to inpatient An order for a bed assignment Start of inpatient admission^5^
Exceptions or exclusions	N/A	ED observation stays, even if later admitted
Incentives	Not slated for inclusion in performance-based payment program; would not be included in overall Hospital Quality Star Ratings	Not slated for inclusion in performance-based payment program; would be included in overall Hospital Quality Star Ratings

Abbreviations: CMS, Centers for Medicare & Medicaid Services; ED, emergency department; N/A, not applicable.

### Age-friendly hospital measure

The CMS HIQR introduced the AFHM in 2025.^3^ As the first structural measure aiming to improve care for older adults, participating hospitals attest “yes” or “no” to statements across 5 domains affirming that they provide care that aligns with the principles of age-friendly care. Domain 3, statement 4, having protocols in place to transfer a targeted percentage of older adults out of the ED within 3 hours of admission or 8 hours of arrival, effectively, sets a 3-hour threshold of harm for boarding of older adults. No boarding process measures are reported as part of this measure.

### Emergency care access timeliness measure

The CMS HOQR requires participating hospitals to report performance on a composite measure, ECAT eCQM.^4^ The ECAT is intended to reduce 4 “access failures” in hospital care, including (1) delayed initial ED evaluation, (2) patients leaving the ED without being seen, (3) prolonged ED length of stay (LOS), and (4) prolonged boarding in the ED. The last component specifies boarding as an inpatient access failure when patients board for greater than 4 hours. Notably, the third component aligns with AFHM in targeting ED LOS over 8 hours. The measure enters voluntary reporting in 2027, with public reporting tied to reimbursement in 2028.^5^ The proposed ECAT measure standardizes boarding times by ED visit volumes and excludes admissions from ED observation units. While pediatric and mental health patients are reported as stratified in reporting, there is no age stratification for older adults. Table 1 compares the AFHM and ECAT CMS quality measures related to hospital boarding.

## Opportunities to revise CMS boarding quality measures

Uniquely, the ED is proximally impacted by regulations that guide both inpatient and outpatient hospital quality programs, which has resulted in misaligned measures targeting the same problem but from different programmatic vantage points. While only ECAT requires reporting of boarding as a process measure, AFHM should evolve to include this type of process measurement as well. As stated in the CMS Meaningful Measures Initiative 2.0, aligning measures across value-based programs has the potential to close measurement gaps and increase efficiency.^6^ Optimizing ECAT and AFHM to incentivize performance begins by aligning how boarding is defined and reported.

The current CMS measures require hospitals to report on boarding using 2 different benchmark thresholds from the same agency, a missed opportunity for standardization. The 4-hour ECAT threshold originated from standards published by the Joint Commission^7^ in 2012, while the 3-hour AFHM threshold was proposed by CMS for the latter measure. ECAT guidance published on the Electronic Clinical Quality Improvement (eCQI) Resource Center stipulates that hospitals choose the earliest time point of 4 possible definitions of decision to admit, the start time to calculate boarding time. The AFHM does not provide equivalent guidance.

Hospital practices vary in what types of patients are considered admitted and thus at risk of boarding. The ECAT excludes patients admitted from ED observation units, since observation is considered an alternative to admission that can reduce boarding and improve care quality and efficiency. Conversely, the Geriatric ED Accreditation Program, in part a model for AFHM, recommends considering ED observation patients as boarders. These conflicting definitions will blur consistent measures and create tension points that will require resolution as voluntary data become available. Standardizing who qualifies as a boarder and how boarding start time is calculated is critical to prevent gamesmanship with reporting and meaningfully compare hospitals.

The ECAT stratifies reporting of boarding metrics by pediatric and adult patients, yet does not distinguish between younger (<65 years) and older (65+ years) adults. The AFHM explicitly targets older adults. While age is far from the only factor in bed prioritization, the documented harms of boarding that disproportionately affect older patients, and the emerging evidence that they are more likely to board, favors the addition of age to bed prioritization algorithms and boarding measures alike. Focusing ECAT's quality measurement on both the youngest and oldest patients by adding geriatric stratification would complement AFHM and prioritize measures that protect older adults, who are more likely to board and are uniquely vulnerable to its harms.

## Conclusion

While CMS policy aspires to ensure cross-coordination and incentive alignment between CMS quality measures, discordant examples remain, impeding the effectiveness of quality measurement programs. By addressing limitations of each measure and aligning them to each other, CMS could maximize their impact on age-friendly care and hospital care access and timeliness, which converge on the patient safety risks of boarding. The ultimate aspirations for CMS policy and each of these measures are to evolve the underlying financial incentives that contribute to hospital boarding and ensuring public accountability. In turn, aligning these measures with each other and tying them to performance-based reimbursement will be critical to eliminating boarding through incentivizing continuous performance improvement.

## Supplementary Material

qxag226_Supplementary_Data
